# Grassland habitat restoration: lessons learnt from long term monitoring of Swanworth Quarry, UK, 1997–2014

**DOI:** 10.7717/peerj.3942

**Published:** 2017-11-14

**Authors:** Barbara Maria Smith, Anita Diaz, Linton Winder

**Affiliations:** 1 Centre for Agroecology, Water and Resilience, Coventry University, Coventry, United Kingdom; 2 Game and Wildlife Conservation Trust, Fordingbridge, United Kingdom; 3 Life and Environmental Sciences, Bournemouth University, Bournemouth, United Kingdom; 4 Primary Industries, Science and Environment, Toi Ohomai Institute of Technology, Rotorua, New Zealand

**Keywords:** Calcareous grassland, Habitat restoration, Seed rate, Seed mix, CSR, Functional signature, Grimes triangle

## Abstract

Habitat restoration projects are often conducted when prior use or extraction of natural resources results in land degradation. The success of restoration programmes, however, is variable, and studies that provide evidence of long term outcomes are valuable for evaluation purposes. This study focused on the restoration of vegetation within a limestone quarry in Dorset, UK between 1997 and 2014. Using a randomised block design, the effect of seed mix and seed rate on the development of community assemblage was investigated in comparison to a nearby target calcareous grassland site. We hypothesised that seed mix composition and sowing rate would influence both the trajectory of the grassland assemblage and final community composition. We found that species composition (in relation to both richness and community assemblage) was strongly influenced by time and to some extent by seed rate and seed mix. However, no treatments achieved strong resemblance to the calcareous grassland target vegetation; rather they resembled mesotrophic communities. We conclude that (as with previous studies) there is no “quick fix” for the establishment of a grassland community; long-term monitoring provides useful information on the trajectory of community development; sowing gets you something (in our case mesotrophic grassland), but, it may not be the target vegetation (e.g., calcicolous grassland) you want that is difficult to establish and regenerate; it is important to sow a diverse mix as subsequent recruitment opportunities are probably limited; post-establishment management should be explored further and carefully considered as part of a restoration project.

## Introduction

The use or extraction of natural resources often results in land degradation, where the ecological value of a site or area is compromised. One approach to mitigate these impacts is to conduct a restoration programme following the cessation of the activity. The success of restoration programmes, however, is variable, and studies that provide evidence of long term outcomes from such schemes are particularly valuable for evaluation purposes (Willis et al., 2007). Long-term monitoring is essential for identifying the time frames required for success (Block et al., 2001) and in order to determine changes in species composition. Meyer et al. (2016), focusing on an 11 year grassland biodiversity study demonstrated that plant diversity effects “*strenghtened over time*”, with increased ecosystem function evident with elevated biodiversity. Hence, the initial establishment of a plant community in a restoration programme is likely to strongly influence ecosystem function as it will influence the trajectory of plant community development.

The debate surrounding the influence of plant diversity on ecosystem function has received much attention (Tilman, Wedin & Knops, 1996; Tilman et al., 1997; Grime, 1997; Hooper & Vitousek, 1997; Grime, 1998; Crawley, Heard & Grant, 1999; Tilman et al., 2001; Turnbull et al., 2016; Gould et al., 2016). Additionally, experimental work has suggested that species richness increases the resilience of grassland communities (Tilman & Downing, 1994; Tilman, 1997a; Naeem & Li, 1997; Del Río et al., 2017). The role of species richness has been linked to more than one ecosystem process. For example, Tilman, Wedin & Knops (1996) suggested that increased species richness leads to increased primary productivity, whilst it also limits nutrient loss from the system (Tilman & Downing, 1994; Symstad et al., 1998). Both productivity and nutrient retention have been cited as important factors in the long term development and stability of a plant community (Loreau, 2000; Wilsey & Potvin, 2000). However, the definition of stability is contentious; a useful resilience-focused definition is “*the capacity of a system to persist in the same state in the face of perturbation*” (Diaz & Cabido, 2001).

Our study focussed on the restoration of chalk grassland within a quarry in the southwest of the UK. We utilised three measures (species richness, species composition and functional composition) in order to evaluate the trajectory of plant communities established at the beginning of the project. Species richness and species composition are generally considered as essential descriptors of biodiversity at a given location, whilst functional composition attempts to identify the contribution of species to ecosystem processes (Tilman et al., 1997). Grime (1977) and Grime (1997) developed thinking around the concept of functional traits, and how these determine the roles of individual species within an ecosystem. His Competitor-Stress Tolerant-Ruderal (CSR) framework has been widely used (particularly in the UK) and allows classification of species with respect to the trade-offs made between reproduction, growth and maintenance (Grime, 1977). Additionally, this approach may be extended and applied to a plant community rather than individual species through the estimation of a “functional signature” (Hunt et al., 2004). The use of this signature approach provides a tool to identify and compare key characteristics of plant communities by concisely representing and comparing functional attributes.

One of the challenges when assessing restoration projects is that success is relative, and will depend on the goals that are defined at the outset of a project (Török & Helm, 2017). It is recognised that confusion frequently arises in the field of restoration ecology as there has been a lack of precision in determining goals (Ehrenfeld, 2000). In addition, there is debate about which level of organization (i.e., individual, species, or community) gives the best measure of the successful establishment (Parker, 1997; Goldstein, 1999; Risser, 1999). In the review by Ehrenfeld (2000), it was suggested that for those engaged in restoration, it is necessary to recognise that a rigid definition of success is *not* achievable. Rather, in most cases, the goals will be site-specific and it is impractical to have the expectation of the exact replication of a selected system. One useful approach applied to the evaluation of restoration projects is the selection of a notional target vegetation as an ecological yardstick by which to test success in re-vegetating an area (Aronson, Dhillon & Le floc’h, 1995; Török & Helm, 2017). This method was used by Stevenson, Bullock & Ward (1995), who selected an area of established chalk grassland close to their experimental site. However, the value of comparing vegetation communities, which are dynamic, is debatable, as each site is unique in terms of context and influences (Pickett & Parker, 1994). Yet comparison of restored grassland with target vegetation at least provides some information on success.

Additionally, the process of restoring grassland on degraded land (such as a quarry which needs to be is the focus of our study) requires consideration of both ecological and financial factors and may be compromised when, for example, financial resources, availability of suitable substrate, or access to local seed material is limited. The use of locally-collected seed is widely recommended for restoration schemes (McKay et al., 2005; Vander Mijnsbrugge, Bischoff & Smith, 2010), but, in practice, often sufficient seed is not available or very expensive to obtain so compromises are needed with regards to both seed rates and seed mixes applied. There is a general lack of long term monitoring of the effectiveness of such widespread “compromised practice” in achieving successful restoration. For example, Stevenson, Bullock & Ward (1995) investigated the effect of three seed application rates in the development of chalk grassland on ex-arable land and initial results found that even a low application rate of locally-sourced species was sufficient to allow for the re-vegetation of large areas. Unfortunately, the experiment was terminated after only two years. The short-time frame of such experiments often precludes arriving at realistic conclusions regarding the success of restoration attempts. This is problematic given the timescale of community establishment; in a review of 40 restored calcareous grassland sites of various ages (Fagan et al., 2008), authors showed that even after 60 years restored grassland species assemblages did not resemble ancient “targets”, although restored sites did move towards targets in terms of function (both ruderality and change to perenniality).

Long-term studies of grassland development will be influenced by both sown species and those that colonize from surrounding vegetation. However, recruitment will be limited by the relative dispersal distances of individual colonizer species and the ability of species to persist (Pywell et al., 2003). Additionally, colonization is also influenced by the plant community being invaded; areas that are more species-rich are assumed to have a more complete use of limiting resources and thus be less easy to invade (Robinson, Quinn & Santon, 1995). In experimental work, Tilman (1997b) found that the greater the initial richness of a site, the more difficult it was for new species to invade. He also discovered that, by adding seeds to an area, species which were best adapted to local conditions at the site colonized readily. In his experiment, a biased subset of available species became established that was based partly on the character of the colonizing species and partly on the number of species already present in the plots. Crawley, Heard & Grant (1999) found that the identity of particular species rather than general species richness controlled the ease with which experimental plots were invaded, and concluded that inter-specific competition was the limiting factor. The study also concluded that seed rate and species composition of the seed mix affected the potential of both calcicolous and weed species to invade at a restoration site.

In our study of the restoration of a chalk grassland on a quarry site, we investigated both seed rate and seed mix, and continued the experiment over an extended time period (17 years) in order to provide additional insights on the destination of community assemblage following establishment. We hypothesised that seed mix composition and sowing rate would influence both the trajectory of the grassland assemblage and final community composition. Specifically, we tested the expectations that:

(i) Increased seeding rates would lead to more rapid and successful development of a plant community appropriate for the location;
(ii) Richer seed mixes would lead to species composition that was more closely matched to adjoining target vegetation;
(iii) That, following establishment, both species and functional composition would vary over time, with progression towards a plant community that had similarity to adjoining vegetation.

## Methods

### Study site

The study was conducted at a limestone quarry in Dorset, UK (NGR SY 970 784) between 1997 and 2014, located on the Purbeck coastal plateau in a landscape of predominantly open farmland that consists of pasture and arable fields. The quarry is bound on three sides by steep sided valleys that support a mosaic of grazed limestone grassland and scrub. Purbeck consists of Jurassic and Cretaceous sedimentary rocks which are principally composed of shales and mudstones, although some sandstone and limestone deposits are present (Portland limestone is extracted from the quarry).

The quarry, initially owned by Tarmac Southern Limited, was in operation throughout the study period. When the operation ceases the quarry as a whole will be landscaped and re-vegetated to resemble nearby limestone grassland. Our field trial was designed to support this objective, and was carried out on an area that had been landscaped in preparation for re-vegetation. Inert materials (comprising stone waste) that had been brought onto site were used to create a gently sloping landform with a plateau on which the experimental plots were laid out. The inert materials were subsequently buried and covered with a minimum of 1 m of the local overburden (i.e., a mixture of clays, marls and limestone chips) that had been stripped from the site before extraction commenced and had been stored on site for this purpose. This material was roughly bladed down, resulting in an uneven surface allowing purchase for the seeds. No topsoil was added and seeds were sown directly onto the undressed surface.

### Experimental design

**Figure 1:**
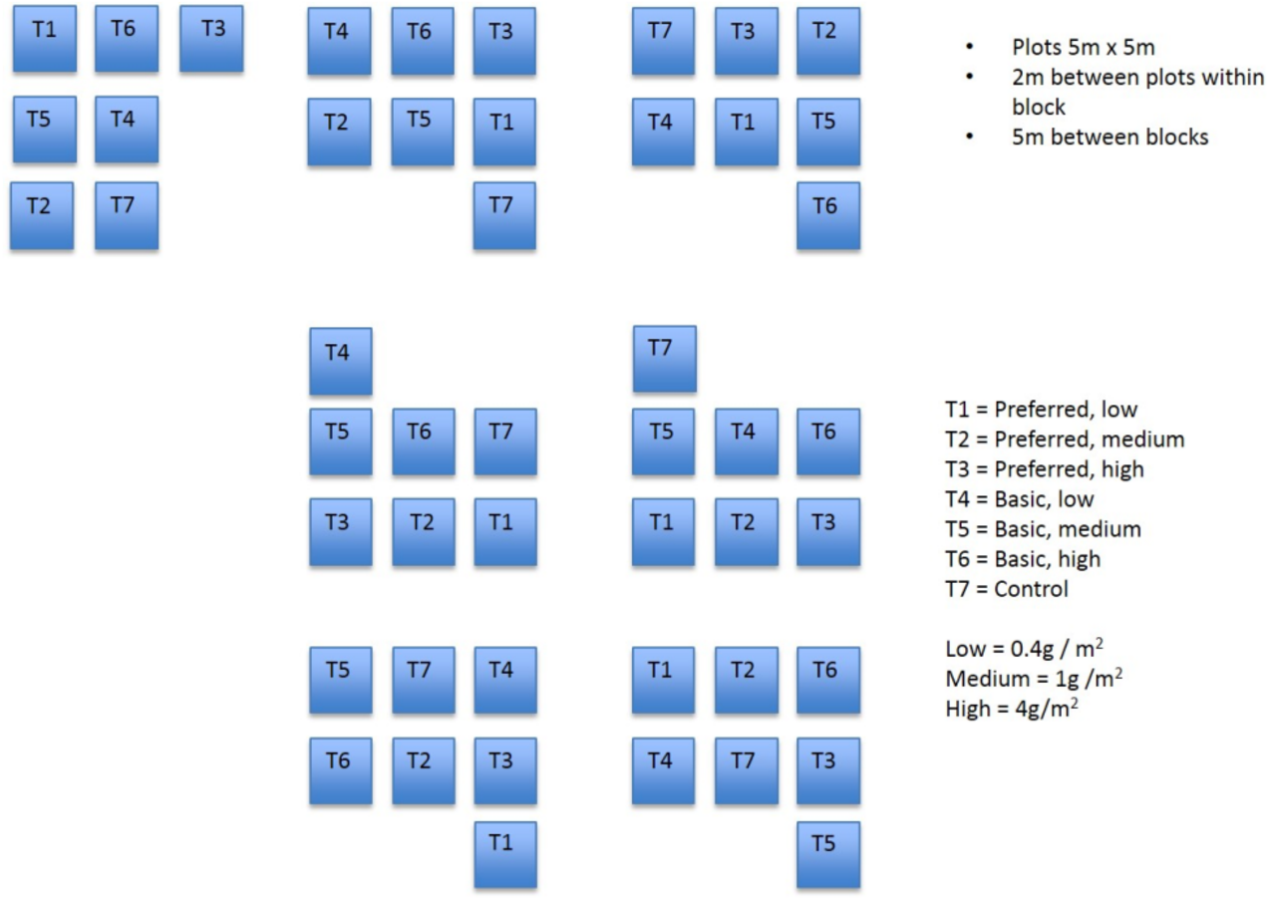
Field plots. Diagram showing layout of field plots.

A randomised block experiment was established at the study site, commencing in March 1997. Prior to the establishment of plots, the substrate was prepared by harrowing and raking but was otherwise untreated. Each of seven blocks comprised seven plots (Fig. 1). Each plot measured 5 by 5 m and the plots were separated by a 2 m buffer. Treatments were allocated randomly to plots within each block.

The UK’s National Vegetation Classification (NVC) system (Rodwell, 1992) was used to define a target plant community and also classify the treatment plots once established. The system is recognised as the “UK standard” and acts as a comprehensive classification and description of plant communities. It covers all natural, semi-natural and major artificial habitats in Great Britain, with communities systematically named and arranged and standardised descriptions for each (http://jncc.defra.gov.uk/page-4259, accessed 10 October 2017). For grasslands, the classification includes mesotrophic, calcicolous, calcifugous, and montane communities which are further categorised into distinct communities.

Treatments included six experimental seed mixes and a control plot. The experimental treatments comprised alternative seed mixes (Table S1), which varied in both species composition (Seed Mix: Basic Mix and Preferred Mix), and application rate (Seed Rate: Low, Medium and High). The Basic Mix comprised 20 species, and was an “off-the-shelf” mixture sold by British Seed Houses (now named Germinal Seeds, http://www.germinal.com). The mix was considered suitable for National Vegetation Classification (NVC) CG2 grassland creation (Rodwell, 1992), which is categorised as a calcareous grassland. The Preferred Mix contained an additional fifteen species that frequently occur in limestone grassland and were recorded in a target site nearby the quarry. Six of these were either known to germinate poorly, or were unavailable as seed, and were therefore introduced as plant plugs. Seed was applied at three application rates; 0.4, 1 and 4 g m^−2^ following practice adopted during the M3 extension project at Twyford Down (Stevenson, Bullock & Ward, 1995). For the Preferred Mix treatments, plant plugs were added at three, five and 15 plants m^−2^ respectively (established at random locations with each plot). The control plots received no application of the basic or preferred mix.

Plots were mown annually during the experiment, from 1999 onwards. In the previous two years the biomass was too low for mowing to take place. Mowing continued throughout the study. A single year of winter grazing with sheep (between September and March) took place in 2004 (sheep wandered freely within an electric fence boundary that enclosed the experimental area). The farmer was unwilling to graze sheep again (he considered it onerous to provide water and check stock). Mowing took place in all years during March (including 2004), and the cuttings were baled and removed.

The target vegetation referred to above was a nearby area considered to be typical of the local calcareous (limestone) grassland. The area (100 m by 100 m), was located within the South Dorset Coast SSSI on a south-east facing slope which supported a mosaic of NVC CG2 and CG4 grassland (Rodwell, 1992). The higher part of the slope was dominated by *Festuca ovina* with a variety of calcicolous grasses including *Anthoxanthum odoratum*, *Briza media*, *Cynosurus cristatus* and *Festuca rubra*, with abundant calcicolous forbs including *Campanula rotundiflora*, *Galium verum*, *Helianthemum nummularium*, *Thymus drucei* and *Viola herta*. The lower part of the slope, which was damp and shaded by trees, was less grazed and in these areas the vegetation was taller, with an abundance of *Brachypodium pinnatum*.

Initially, the experiment was also established incorporating split-plots to investigate the effect of a nursery grass on establishment. Wells, Bell & Frost (1981) recommended that the annual grass *Lolium multiflorum* be used as a nurse grass as it is unlikely to persist. However, when applied, this species failed to establish, so the experiment was continued without this design element. Data from each plot was consolidated so that this factor was not included in any analyses.

### Recording

Recordings were done in the summers of 1997, 1998, 2000, 2003, 2007 and 2014. Within each plot, species cover was recorded within a 1 m × 1 m quadrat leaving 0.5 m at the margins to avoid edge effects. The quadrat position was marked with pegs so that in each subsequent sampling year the same area of vegetation could be recorded. Cover was recorded by visual estimates of the percentage cover for each species. During the period 1997–2007 all seven blocks, and in 2014 three blocks, were surveyed respectively. In 2007 and 2014 quadrats within the target vegetation were also recorded for comparative purposes (seven permanent plots were recorded within the target area on each occasion). For all years, species recording was supervised and led by the same botanist.

### Data analysis

Data was explored using both simple univariate and multivariate approaches. Summary statistics of species richness (and standard error), mean percentage cover and percentage of replicates in which a species was observed (Tables S3 and S4) were calculated. Preliminary analyses indicated that all controls were substantially different from treatments. We therefore chose to exclude the control plots from statistical tests in order to avoid trivial significant outcomes that did not address the central question of the study which related to the establishment of vegetation. This approach also facilitated the adoption of a fully factorial analysis approach. However, control data were included in graphical summaries when appropriate for comparative purposes.

Initially, for each sampling year, species richness of treatments were compared using analysis of variance, with Seed Mix and Seed Rate as fixed factors, Block as a random factor and a Mix*Rate interaction factor. Richness was log_10_(*n* + 1) transformed prior to analysis and the Minitab (version 17) GLM function was used. Trends in species composition between years and treatments were investigated using both Non Metric Multidimensional Scaling (nMDS) and Permanova (Primer 7). For nMDS, mean percentage cover was calculated for each treatment for each year and used to generate a simple summary plot that could be evaluated visually. Data were square root transformed and Bray Curtis similarity indices calculated. Additionally, for each year separately, Permanova was done to investigate differences in species composition between treatments. A three factor design (Seed Mix, Seed Rate, Block) with a Mix*Rate interaction term was adopted using Type III sum of squares to investigate whether Seed Mix and Seed Rate influenced species composition. Data were square root transformed prior to analysis.

Analysis was then done to investigate shifts in the community functional traits over time. Using the summarised data from the initial nMDS analysis, each species was classified following Grimes’ C-S-R functional classification (Grime, Hodgson & Hunt, 1988) following the classification adopted by Hunt et al. (2004). Using this classification, 19 “pseudo-species” were generated which represented each functional classification (i.e., C, C/CR, C/CSR, C/SC, CR, CR/CSR, CSR, R, R/CR, R/CSR, R/SR, S, S/CSR, S/SC, S/SR, SC, SC/CSR, SR, SR/CSR) by calculating the total percentage cover of species represented within each category. These data were then explored using nMDS to determine similarities in functional composition between years and treatments. Further, the functional signature was calculated for each treatment and year following the method of Hunt et al. (2004). Species were omitted from the analysis if they did not have a CSR category assigned within the Hunt et al. (2004) classification (those species included are identified in Tables S1 and S2). This method, for a given sample, concisely represented the functional attributes present amongst component species.

To determine how treatment and time influenced the presence of individual species, redundancy analysis (RDA) was conducted (Canoco Version 4.5). In this analysis, the influence of explanatory variables on species composition was explored. Year, Seed Rate, and Seed Mix were included as explanatory variables and a manual stepwise selection process was adopted that enabled inclusion of parameters that significantly influenced species composition. Seed Rate and Seed Mix were included as dummy variables as required by the Canoco system. Cover estimates were square root transformed. A biplot was used to graphically represent these relationships (Canodraw Version 4.0).

Finally, comparisons between the treatments and the target vegetation plots sampled in 2007 and 2014 were made by evaluating the NVC category designations for each treatment. Constancy values (I–V) were calculated from the observed frequency of species during the study, and NVC category was calculated using MAVIS Plot Analyser (Version 1.3, Centre for Ecology and Hydrology).

## Results

In total, 149 species were recorded during the experimental study (Tables S1 and S3), whilst 51 species were recorded at the reference site (Tables S2 and S4). The reference site comprised a range of characteristic calcicolous forbs including *Asperula cynanchica*, *Cirsium acuale*, *Campanula rotundiflora , Carlina vulgaris, Euphrasia nemorosa, Helianthemum nummularium*, *Ononis repens* and *Scabiosa columbaria.* Grasses indicative of calcicolous conditions were also present including *Brachypodium pinnatum*, *Briza media*, *Cynosurus cristatus*, and *Festuca ovina*. There was a decline in species richness between 2007 (42) and 2014 (29) on the target area.

During the experiment, some species were very commonly recorded, including *Achillea millefolium*, *Agrostis stolonifera*, *Anthoxanthum odoratum*, *Anthyllis vulneraria*, *Arrhenatherum elatius*, *Briza media*, *Centaurea nigra*, *Cynosurus cristatus*, *Dactylis glomerata*, *Daucus carota*, *Festuca ovina*, *Festuca rubra*, *Galium verum*, *Holcus lanatus*, *Leontodon hispidus*, *Leucanthemum vulgare*, *Lotus corniculatus*, *Medicago lupulina*, *Pastinaca sativa*, *Picris echoides*, *Plantago lanceolata*, *Plantago media*, *Poa pratensis*, *Primula veris*, *Prunella vulgaris*, *Ranunculus repens*, *Rumex crispus*, *Sanguisorba minor, Trifolium repens* and *Triseum flavescens*. These species were recorded consistently across multiple years and treatments. We observed no consistent pattern with regards to the success of plug plants compared to those established by seed. However, there was an indication that the use of plug plants at least promoted initial establishment (Table S3), although this did not consistently result in the long term presence of those species.

**Figure 2:**
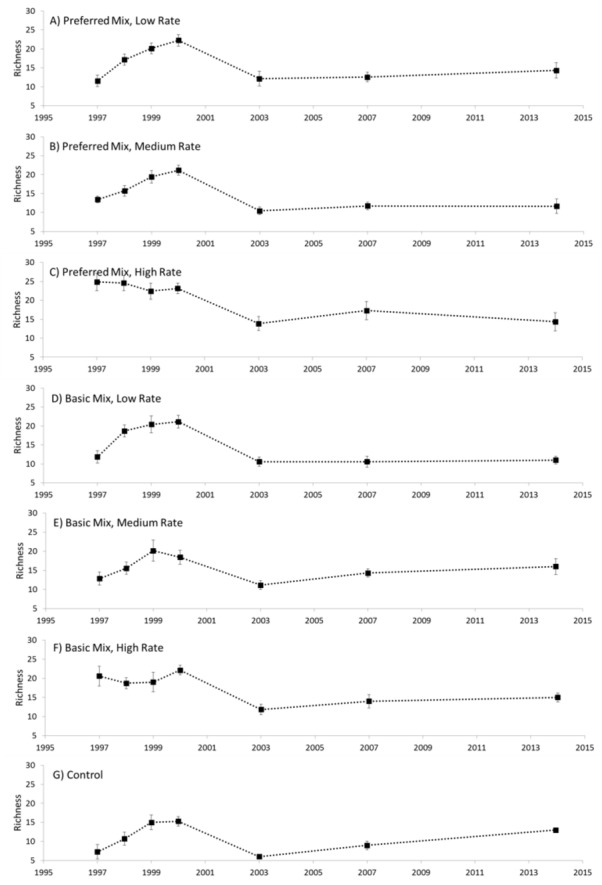
Species richness. Species richness (mean ± 1 s.e.) recorded for each treatment (A–G), 1997–2014.

Distinct changes in richness were evident during the experimental study (Fig. 2). In general, richness increased from the start of the experiment in 1997, peaking in 2000 and then declining to a relatively low value in 2003. Between 2003 and 2014 there was a general and gradual increase in richness evident in both the experimental treatments and control. Analysis of variance revealed that Seed Rate influenced richness to some extent, particularly at the start of the experiment in 1997 and 1998 (Table 1), where richness increased with higher seed rates. However, this effect did not persist beyond 1998 when a rapid increase in richness was evident in the low and medium rate plots. Seed Mix had no measurable effect on richness, and no interaction was evident. We also noted that the communities established in 1997 were generally similar for all seeding rates, but, in 1998 the low and medium rates diverged from the high rate application.

**Figure 3:**
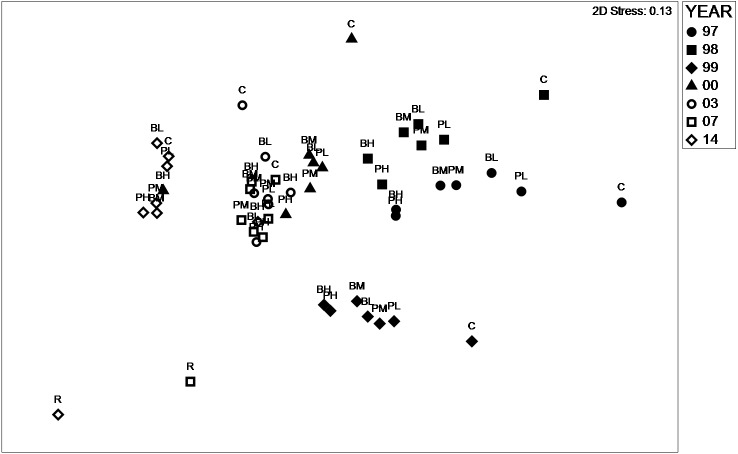
nMDS plot similarity species. Non-metric multidimensional scaling plot representing similarity between treatments 1997–2017 based on species assemblages. B, Basic Mix; P, Preferred Mix; L, M, H, Low, Medium and High rates respectively. C, control; and R, reference.

**Table 1:** ANOVA richness. Summary statistics for analysis of variance of species richness by year. *F*-values and associated significance shown (* *P* < 0.05, ** *P* < 0.01, *** *P* < 0.001). For 1997–2014, *d.f.* were: Mix = 1, 30; Rate = 2, 30; Rate*Mix = 2, 30; Block = 6, 30. For 2014, *d.f.* were: Mix = 1, 10; Rate = 2, 10; Rate*Mix = 2, 10, Block = 2, 10.

Year	Mix	Rate	Rate*Mix	Block
1997	1.2	22.4***	0.6	0.02*
1998	1.0	8.2**	2.7	2.4
1999	2.2	0.3	2.3	20.6***
2000	1.9	2.0	0.3	0.9
2003	0.8	1.8	0.8	8.2***
2007	0.5	3.5*	2.2	2.6*
2014	0.2	0.6	2.0	0.1

Analysis of species assemblage using nMDS (Fig. 3) revealed that the similarity between species assemblage was primarily dependent on Year, although Seed Rate and Seed Mix had some influence within a year. The stress value (2D) of 0.13 was considered acceptable. In 1997, 1998, 1999 and 2014 both the experimental treatments and controls were represented as distinct clusters. In 2000, 2003 and 2007 experimental treatments and controls were also distinctly clustered, although they overlapped indicating relative similarity when compared to other years. Controls were associated with each year cluster, but, their position on the plot indicated that they were generally quite dissimilar from the experimental plots. The reference site sampled in 2007 and 2014 was distinctly dissimilar from the other plots. In addition to the between-year differences evident from the nMDS plot, Permanova analysis focused on within-year comparisons and indicated that the treatments were measurably different in their species composition, with the exception of the 2014 (Table 2). Both Seed Mix and Seed Rate significantly influenced assemblage, but, no interaction between these factors was evident. Inspection of the nMDS plot also revealed a reduction in within-year dissimilarity between 1997 and 2014, with the cluster size clearly reducing during the study.

**Figure 4:**
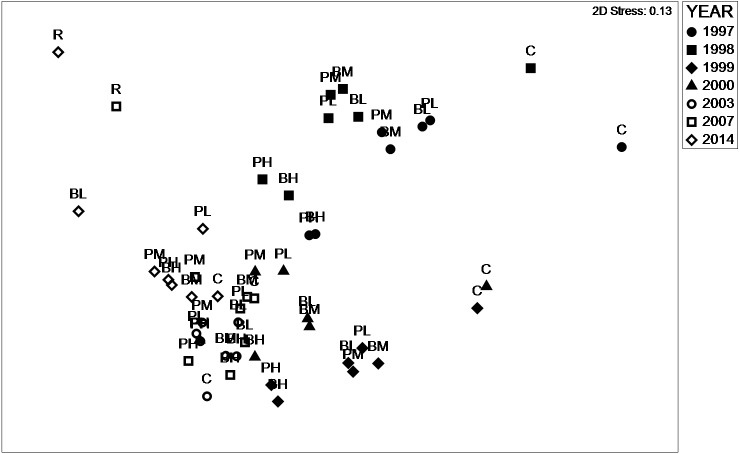
nMDS functional psudospecies. Non-metric multidimensional scaling plot representing similarity between treatments 1997–2017 based on functional pseudospecies.

nMDS based on functional pseudo-species generally revealed a similar pattern to the species-based analysis (Fig. 4). The stress value (2D) of 0.13 was considered acceptable. In 1997, 1998, 1999 and 2014 experimental treatments formed clusters, although they were less distinct than the species-based analysis. This clustering was also evident in 2000, 2003 and 2007 albeit with overlap between the years. These results indicated that differences in community assemblage between years was characterised by both species composition and the functional role of those species. As with the species-based analysis, the target site was substantially different to the experimental plots, indicating that this semi-natural habitat had distinct functional characteristics.

**Table 2:** Permanova species. Summary statistics for Permanova of species assemblage by year. Pseudo *F*-values and associated significance *P*(perm) shown (* *P* < 0.05, ** *P* < 0.01, *** *P* < 0.001). All Permanova were completed with 996–999 permutations. Degrees of freedom follow those summarised in Table 1.

Year	Mix	Rate	Rate*Mix	Block
1997	7.1***	1.8*	0.83	2.8***
1998	6.6***	4.8***	0.87	3.3***
1999	5.8***	6.3***	1.1	4.2***
2000	4.8***	8.2***	1.3	2.4***
2003	1.8*	5.3***	1.7	3.2***
2007	2.3***	2.5**	1.4	3.1***
2014	0.86	0.49	1.0	1.8

**Figure 5:**
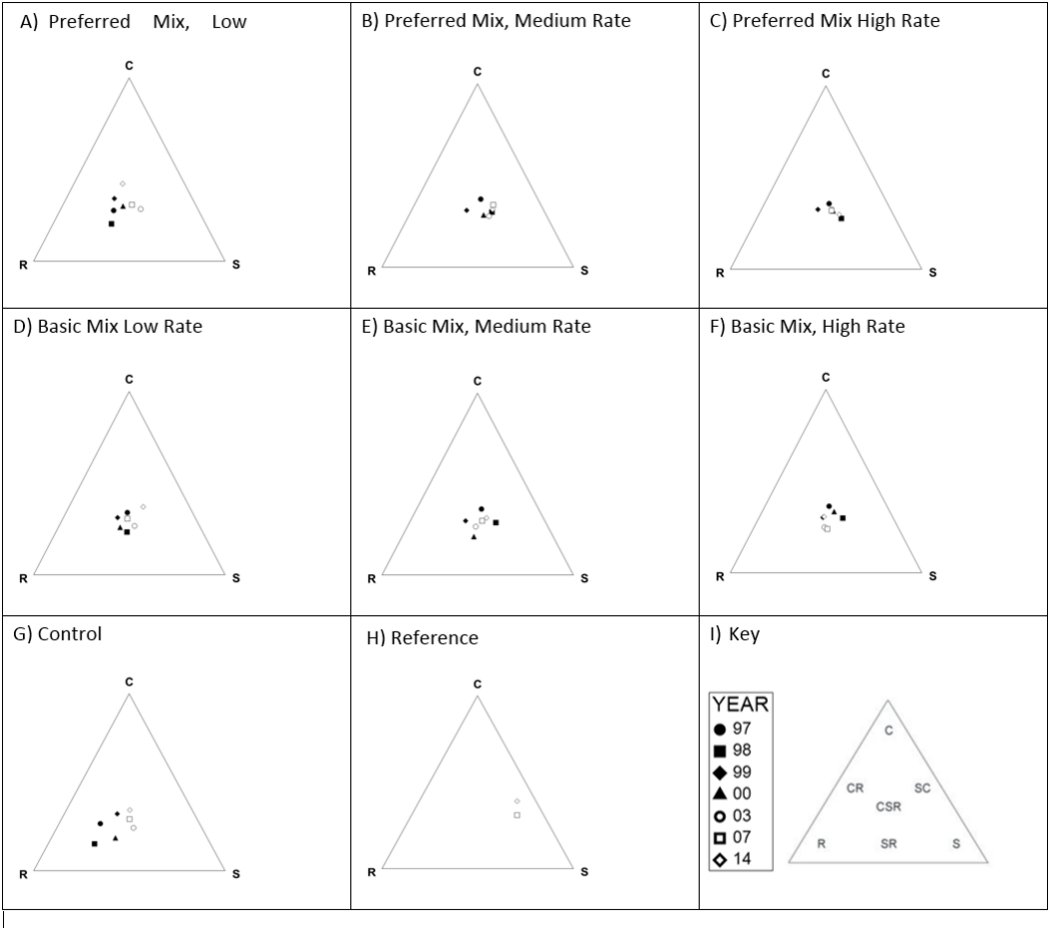
CSR functional signatures. Functional signatures for each experimental treatment, control and reference site 1997–2017 following method of Hunt et al. (2004). Each diagram (A–H) represents the functional signature for the given experimental treatment recorded for each sample year. Key to symbols representing each year are provided in (I), which also indicates the location of each functional signature within the triangle.

Characterisation of the functional signatures of these data (Fig. 5) indicated that the experimental treatments were predominantly in the CSR region (Hunt et al., 2004), whilst the controls were closer to the R region. The target site had an SC-S functional signature, which was substantially different from all experimental treatments and controls. No clear progression in functional signature was evident during the study, with the possible exception of the control plots which shifted from being strongly R to a CSR position.

**Figure 6:**
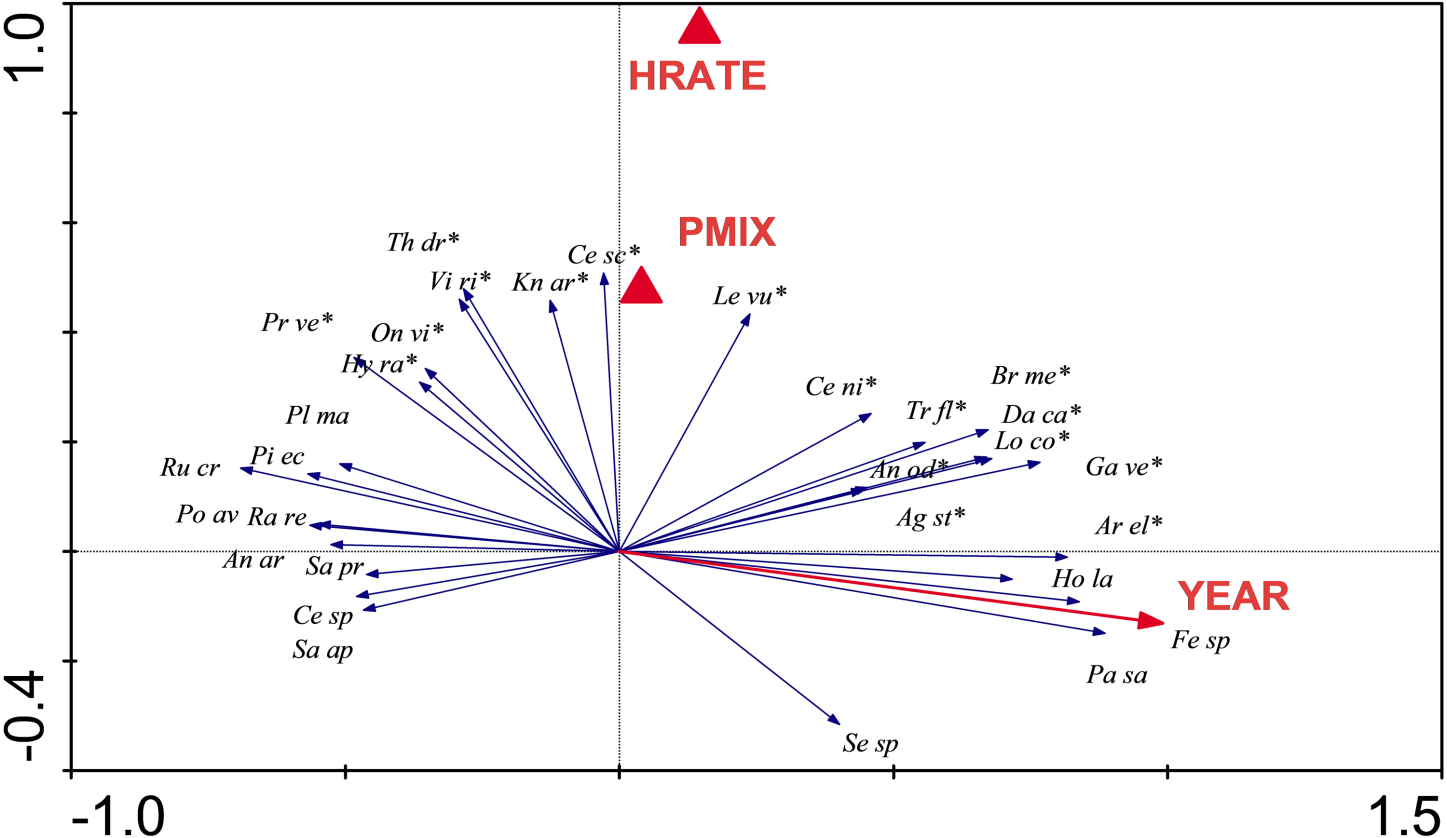
RDA analysis. Redundancy Analysis showing the relationship between individual species and statistically significant experimental parameters. Only the thirty most strongly influenced species are shown for the sake of clarity, and those that were included in original seed mixes are indicated by an asterisk. RDA requires that factors are coded as dummy variables (indicated as filled triangles); HRATE, high Seed Rate and PMIX, Preferred Mix. The YEAR arrow indicates the trajectory of changes in species composition that are influenced through time.

Redundancy analysis showed species composition was strongly influenced by Year (*P* = 0.002), and to a lesser extent by High Rate (*P* = 0.016), and Preferred Mix (*P* = 0.04). The first two axes of the ordination described 25.5% of variability in species composition (Fig. 6). Some species increased in abundance during the trial. For example *Arrhenatherum elatius*, *Festuca* spp., *Holcus lanatus* and *Pastinaca sativa* increased and are typical of mesotrophic grassland communities. Other species decreased, such as *Picris echoides*, *Plantago major*, *Polygonum aviculare* and *Rumex crispus*. The latter species are early colonising, ruderal species and their decline was consistent with a closing sward dominated by grasses. Species that were dependent on Seed Rate or Seed Mix included *Centaurea scabiosa*, *Knautia arvensis* and *Leucanthemum vulgare*, all introduced as part of the experimental seed mixes (Table S3).

Finally, NVC classification indicated that none of the experimental treatments (or controls) were classified as equivalent to the reference site (Table 3). The experimental treatments were predominantly classified as neutral or mesotrophic grasslands (MG), generally characterised by grasses and herbs with a pH range of 5 to 6.5 (Natural England, 2007). The reference sites were calcicolous grassland (CG), generally characterised by a pH range of 6.5–8.5.

**Table 3:** NVC categories. NVC classifications for target site, experimental treatments and control for 2007 and 2014. P, preferred mix; B, basic mix; L, M, H represent low, medium and high seed rates respectively. Data shown includes three highest NVC classifications estimated using MAVIS and associated percentage fit.

	Target	PL	PM	PH	BL	BM	BH	Control
***2007***								
1.	CG4b 54	MG5 48	MG1d 49	MG5b 54	CG6 46	MG5b 53	MG1d 52	SD8a 47
2.	CG4 53	MC11 48	MG1 49	MG5 52	MG5 45	CG4c 51	CG4c 51	MC11 44
3.	CG3b 50	MG5b	MC11 48	MG5a 51	CH4c 45	MG5 50	CG4 50	MG6 44
***2014***								
1.	CG4b 47	MG5b 45	MG1d 46	MG5b 44	MG1d 43	MG5b 49	MG5b 47	MG1d 40
2.	CG5a 47	MG1d 44	CG4c 45	MG1e 44	MG1a 39	MG5 45	MG1e 46	SD8a 39
3.	CG4 44	MG1e 42	MG1 43	MG1d 43	MG1 39	MG1e 45	MG5 45	MC9c 39

## Discussion

This study investigated how choices regarding the initial establishment of a plant community influenced the long-term outcome of a habitat restoration project, and focussed on determining the impact of seed mix and sowing rate. Such studies provide data useful in guiding the expectations of policy makers and land owners with reference to: (i) the potential biodiversity outcomes; and (ii) time-frame. Our aim was to establish a diverse sward, typical of calcareous (limestone) grassland, by sowing a seed mix that included known calcicoles.

Our “measure of success” was similarity to a target habitat of established limestone grassland in a neighbouring valley within 500 m of the experimental area. We selected our species to be established by comparison with this site, and also recorded species composition of the target site in 2007 and 2014. In retrospect we should have surveyed the target site during the entire duration of the study, particularly as there appeared to a shift (decline) in richness at the target site; a reflection of the dynamic nature of plant communities. Our experimental plots did not match the target vegetation well, and this is possibly not surprising given the 60 year-plus timescale of grassland restoration (Fagan et al., 2008).

We observed substantial shifts in species composition that were mediated by both time and restoration treatment. We also observed substantial differences between the blocks of the experiment, which was probably due to a combination of inconsistencies in substrate and aspect of the blocks themselves. After 17 years, however, none of the experimental treatments approximated limestone grassland as exemplified by the target habitat. Using the UK’s National Vegetation Classification we observed that, after 17 years, the experimental treatments (and controls) were considered to be Mesotrophic Grassland (MG) compared with the target habitat which was classified as Calcicolous Grassland (CG). The failure to approximate the target habitat has been observed in other studies (Gilbert, Gowing & Bullock, 2003; Sternberg et al., 1999). Long-term monitoring is essential for identifying the time frames required for success (Block et al., 2001), and any pivotal processes such as ecological tipping points (Brook et al., 2013) and impact of changes in diversity. Although we did not specifically investigate the idea of a tipping point, there was a consistent point in time (2000) where a shift in richness occurred (Fig. 2). Further study of the idea of tipping points during restoration may therefore be warranted and fruitful.

Species recruitment has been shown to be strongly dispersal-limited (Bakker & Berendse, 1999). This observation is supported by our work; in the study the 25 m^2^ plots, separated by two metres, remained distinct for six years and after fifteen years there was little recruitment of additional unsown calcareous species from the target habitat located within 500 m. Therefore, additional sowing with locally sourced target species may be beneficial (Baasch et al., 2016). The use of plug plants, which promote initial establishment, may be particularly valuable. Additionally, the continued paucity of species on the control plots up to and including 2007 demonstrated that natural regeneration unassisted by sowing is unlikely to be a useful approach in the case of calcareous grassland. The timescale of community establishment should be considered when developing outcomes and objectives for such projects; elsewhere it has been suggested that the natural regeneration of grassland communities (unsupported by seed sowing) could reasonably be expected to take a century (Redhead et al., 2014). This underlines the importance of long-term monitoring so that such projects are meaningfully evaluated. This is, however, a continuing challenge for ecologists as projects rarely include monitoring beyond five years, although individual researchers may return to re-evaluate sites (Fagan et al., 2008).

During the experiment the plots, in general, initially increased in richness, with a subsequent decline and gradual increase. The decline in species generally resulted in richness that was lower at 15 years than immediately after sowing. This may be attributed to the initial establishment of mainly ruderal species or arable weeds in the early phases of the study (including *Kickxia elatine*, *Polygonum aviculare*, *Rumex crispus*, *Sagina apetala*, *Sinapis* sp, and *Tussilago farfara*) and the failure of sown species to establish. Species that were included within the seed mixes but did not persist included *Hypochaeris radicata*, *Medicago sativa*, *Rhinanthus minor* and *Viola riviniana*. Two species that were sown, but, failed to establish were *Alliaria petoilata* (more commonly found on acidic soils) and *Helianthemum nummularium*, a typical member of the calcareous community but dependent heavily on management for germination that is known to persist poorly (Poschlod, Hoffmann & Bernhardt-Römermann, 2011; Pywell et al., 2003). These results provide evidence of a possible very early tipping point in the restoration process where the trajectory is diverted away from success by early competition. It provides support for wider concerns about tipping points (Brook et al., 2013) and further indicates the importance of long-term monitoring of other restoration to identify and mitigate against tipping points that lead to unsuccessful restoration outcomes.

By 2003, six years after the trial started, richness was similar across all the experimental treatments, whilst the control plots had lower richness. This was perhaps surprising given the proximity of the plots; it was not until 2014 that the control had richness that approximated to that of the experimental treatments. It is unfortunate that similar studies have been stopped (e.g., Stevenson, Bullock & Ward, 1995) as authors considered the experiment to be confounded; we found that over an extended time period differences remained evident between plots close to each other indicating that dispersal is a major constraint when plant communities are re-established. Öster et al. (2009) recognised that dispersal strongly limits recruitment (albeit mediated by establishment opportunities). Community establishment is clearly long and needs to be assessed over decades rather than years. The expectation of rapid and successful community establishment is largely misguided.

None of the treatments closely resembled the target community, although this is perhaps unsurprising given that the age of the target community (c. hundreds of years) compared to the “young” communities following the restoration. Investigation of the functional signatures of the plant communities indicated that they were positioned generally within the CSR domain. With the exception of the control, there was no clear progression/change in functional signature over time for the treatments. The control, however, clearly shifted from a ruderal signature to one more akin with the experimental treatments. We attribute this to the initial establishment of ruderal species and then the colonisation of species adjacent areas and experimental plots. The target habitat clearly occupied a different niche (SC), lacking the ruderal species that persisted in the experimental plots.

The RDA illustrated substantial species turnover. Ruderal species were evident at the start of the experiment, but, abundance of these declined over time (e.g., *P. aviculare*, *Sinapis* sp. and *V. persica*). More grassland-typical species became established over time. Lack of grazing at the site (which was not possible as the site was within a working quarry) meant that perennial grasses dominated over time, probably reducing the availability for microsites which would give opportunities for less competitive species to spread (Sternberg et al., 1999). Although a cutting regime was introduced to mimic grazing, mowing does not provide the microsites for the establishment of forbs that grazing does. Further studies could experimentally test how long-term management regimes (e.g., mowing, grazing, etc.,) influence the outcomes of grassland restorations.

Any of the typical forbs that comprise limestone grassland are known to be poor competitors while generalist species are known to perform better in dense vegetation (Pywell et al., 2003). *Rhinanthus*, which is known to control grasses, failed to persist at the site and emphasises the importance of management. Grazing not only provides microsites but also reduces the build-up of biomass. Biomass build-up leads to an increase in organic matter that will increase fertility and drive the site to a mesotrophic system, despite the underlying low fertility of largely limestone chip in local clay overburden that was the primary substrate. Biomass build up, if it occurred, could shift the species assemblage away from the target calcareous community. In our study, grasses dominated and created a dense sward that probably restricted sites for calcareous species to establish, which was likely exacerbated by the lack of grazing animals. Such grasses mat and rot down, creating a relatively nutrient-rich soil which direct the community onto a mesotrophic trajectory. Our management regime of annually mowing may simply not have removed sufficient biomass to drive changes in nutrient levels. A “mesotrophic” tipping point (Brook et al., 2013) may occur early, in which case management intervention could redirect community development. This early management may be crucial as once mesotrophic grassland is established it is very difficult to shift towards a calcareous community.

We conclude that: (i) as with previous studies, there is no “quick fix” for the establishment of a grassland community; (ii) that long-term monitoring provides useful information on the trajectory of community development; (iii) that sowing gets you something (in our case mesotrophic grassland), but, it may not be the target vegetation (i.e., calcicolous grassland) that is difficult to establish and regenerate; (iv) it is important to sow a diverse mix as subsequent recruitment opportunities are probably limited (high rates of sowing and the preferred mix influenced composition; Fig. 6); and (v) post-establishment management should be carefully considered as part of a restoration project.

##  Supplemental Information

10.7717/peerj.3942/supp-1 Table S1 Raw data experiment Values represent percentage cover.

10.7717/peerj.3942/supp-2 Table S2 Raw data for reference site Values represent percentage cover.

10.7717/peerj.3942/supp-3 Table S3 Summary data for experimental treatments Main values represent mean percentage cover. Values in brackets represent percentage of plots where species was recorded.

10.7717/peerj.3942/supp-4 Table S4 Summary data for reference site Main values represent mean percentage cover. Values in brackets represent percentage of plots where species was recorded.

## Additional Information and Declarations

### Data Availability

The following information was supplied regarding data availability: The raw data has been provided as a Supplemental File.
